# A multi-centre quality improvement project to assess the impact of a standardised NIV care bundle on mortality outcomes in patients with acute type 2 respiratory failure

**DOI:** 10.1016/j.clinme.2026.100574

**Published:** 2026-05-06

**Authors:** Christopher J. Michie, Kirsty R. Ward, Peter W. Creber, Susie Beresford, Mark C. Juniper, Rebecca Helen Mason

**Affiliations:** a Royal United Hospital Bath, United Kingdom; b North Bristol Southmead Hospital, United Kingdom; c Health Innovation West of England, United Kingdom; d Great Western Hospital, United Kingdom

**Keywords:** Respiratory, COPD, Obesity hypoventilation, Neuromuscular disease, NIV, Acute, QI, Mortality, Care bundle, NCEPOD, BTS

## Abstract

The evidence for non-invasive ventilation (NIV) is clear; however, real-world outcomes fall short of those demonstrated by clinical trials. We developed a five-step care bundle in line with BTS quality standards to standardise management and guide clinicians through the first few hours of NIV care. This was combined with staff training initiatives. The project aimed to reduce acute NIV mortality to 10%. Although this target was not met, the project delivered a 7% reduction in mortality from 28% to 21%, equating to 143 fewer deaths in 2023 compared to 2022. The project also delivered increased staff confidence. Through this project we have demonstrated that early, effective NIV, in appropriate patients, delivered in a standardised way by confident and competent staff, improves outcomes.

## Introduction

Non-invasive ventilation (NIV) is a well-established, life-saving intervention for acute hypercapnic respiratory failure (AHRF). A seminal randomised controlled trial (RCT) demonstrated that NIV reduced mortality to 10% in patients with COPD.^1^ Subsequent British Thoracic Society (BTS) audits reported increasing mortality rates reaching 34% in 2013,^2^, ^3^, ^4^ prompting the 2017 National Confidential Enquiry (NCEPOD) report, identifying several areas for improvement.^5^

BTS quality standards for acute NIV were published in 2018,^6^ leading to a slight reduction in mortality in recent audits; however, mortality remains higher than other countries with equivalent healthcare systems.^7^, ^8^, ^9^

The primary project aim was to reduce mortality to 10% or lower in patients receiving acute NIV. A five-component care bundle based on BTS quality standards was agreed (Appendix-1). The bundle focused on decisions taken in the first few hours of treatment. The components of this bundle and brief rationale for each are listed below:

• **Appropriate case selection:** inappropriate patient selection significantly contributes to worse real-world outcomes.^10^ NIV should only be delivered to patients in AHRF with an evidence-based indication.^6^
• **Treatment escalation plan in place:** a discussion should be completed with specific reference to suitability (or not) for invasive ventilation.
• **NIV to be started within 60 min of decision to treat:** prolonged acidosis worsens prognosis. The NCEPOD review found that NIV was delayed in 27% of patients and the National Respiratory Audit Programme showed that only 21% of patients with AHRF commenced NIV within 2 h.^5^, ^11^
• **Inspiratory pressure (IPAP) of 20 cmH₂O to be achieved within 60 min:** insufficient inspiratory pressure will not augment ventilation sufficiently to correct respiratory acidosis. A target IPAP was agreed in line with BTS guidance.
• **Repeat arterial or capillary blood gas within 2 h of starting NIV:** the objective of NIV is to correct respiratory acidosis. Repeat blood gases are essential to monitor the effectiveness of the treatment.

## Methods

The project was conducted across six hospitals in the west of England. Staff confidence was assessed using questionnaires at the start of the project and at 1 year. The bundle was introduced across the region in January 2023. Data from January 2022 to January 2023 established baseline mortality. Data were collected in parallel with deployment of the bundle in line with the Model for Improvement framework.

The primary outcome, mortality rate, was calculated monthly from all patients coded as receiving acute NIV who died during that hospital admission, divided by the total number receiving acute NIV that month. The mortality rate from all trusts was combined and reported monthly.

The following process measures were assessed: use of the bundle, appropriate NIV, commencement within 60 min, IPAP escalation, and repeat blood gas within 2 h. These were evaluated retrospectively by clinicians involved in NIV care from a random sample of two patients per hospital, per week, identified after discharge by the business intelligence unit coded as having had NIV. This equated to 346 patients. Data were anonymised and from routine parameters, therefore patient consent was not required.

## Results

### Staff confidence

Clinicians involved in acute NIV care (n = 213) completed the survey at the beginning of the project and again at the end of the first year (n = 111). Most respondents worked in respiratory departments. More than a third worked in acute-care areas where NIV is usually started.

**Fig. 1:**
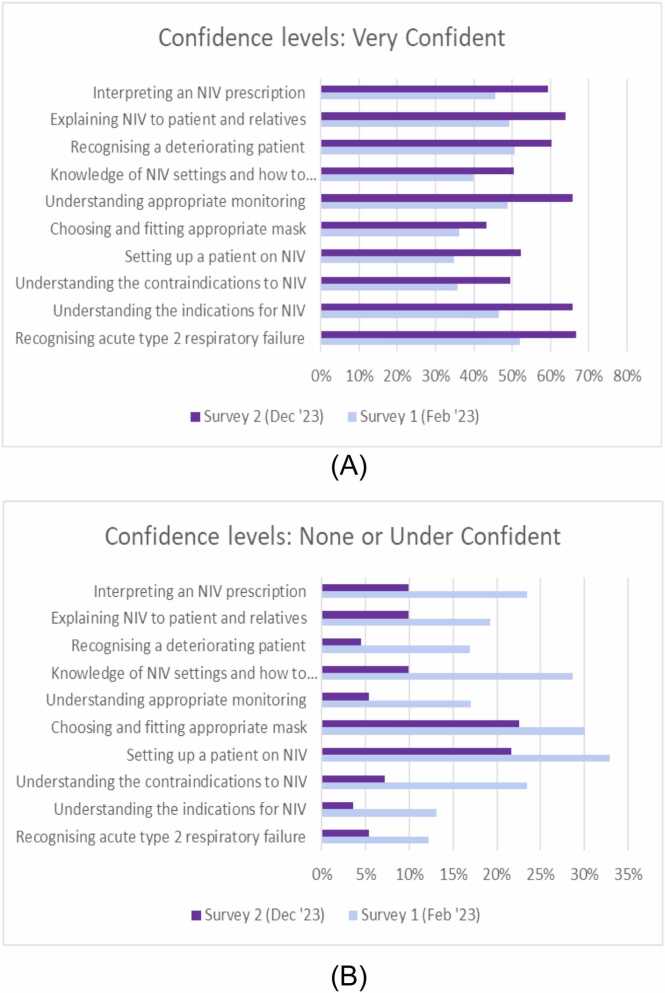
A and B: Staff confidence in use of acute NIV pre- and post-intervention.

The percentage of staff rating themselves very confident increased in all domains between the surveys (Fig. 1A). Similarly, the percentage of staff rating themselves having no confidence or under-confident decreased (Fig. 1B).

### Process measures

**Fig. 2:**
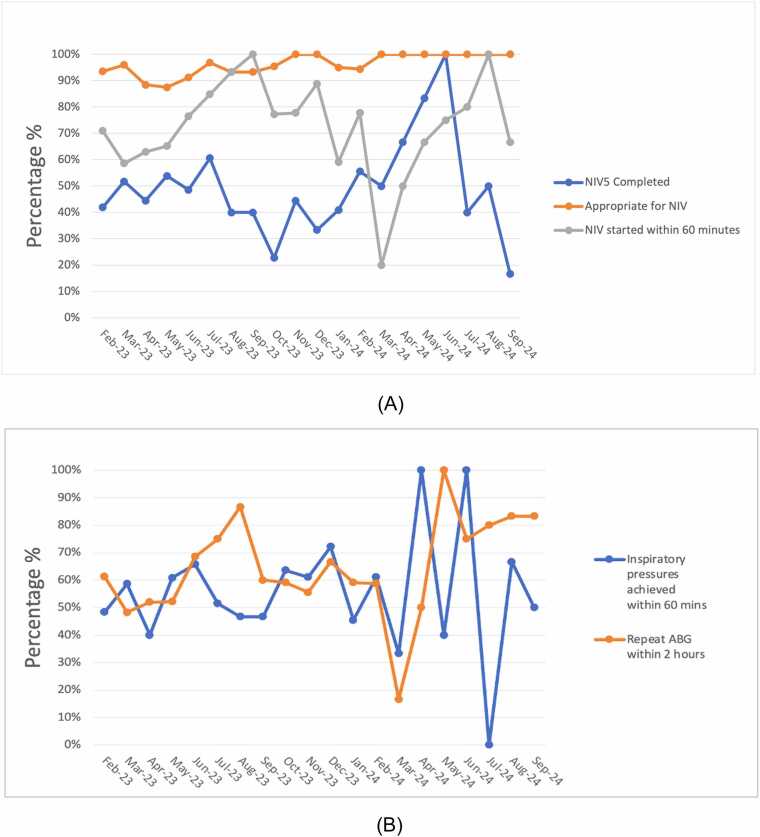
A and B: Process measures February 2023–September 2024.

Bundle use was inconsistent, with slight improvement over time. Fig. 2A demonstrates improvement in appropriate use of NIV to 100% for the last 7 months. Appropriate indication for NIV was retrospectively assessed in 333 sample patients; in 290 cases NIV was considered appropriate; of those, 75% survived, compared to 55% in the inappropriate group. IPAP escalation to 20 cmH20 was variable, but reached 100% in 2 months. Repeat blood gas within 2 h of starting NIV improved over time.

### Mortality data

**Fig. 3:**
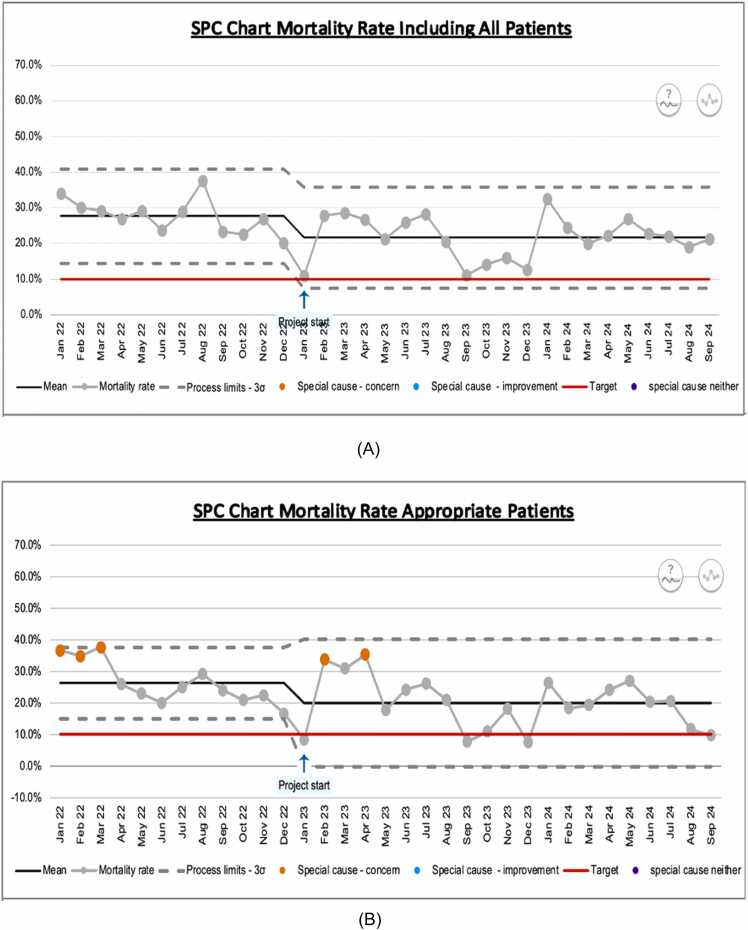
A: Mortality rate SPC chart (all patients) January 2022–September 2024. B: Mortality rate SPC chart (appropriate NIV patients only) January 2022–September 2024.

The SPC chart (Fig. 3A) demonstrates that the baseline mean mortality rate was 28%. Post-intervention, this fell to 21%. The target mortality of 10% was not achieved. However, the 7% reduction in mortality equated to 143 fewer deaths in 2023 compared to 2022. The target mortality rate was initially outside the control limits, demonstrating that the system was not designed to deliver such a low mortality rate. After bundle introduction, the 10% target was within control limits, suggesting that achieving this target is possible with further improvements.

When only patients retrospectively deemed appropriate were included in the analysis, reduction in mean mortality was similar (Fig. 3B). However, mortality in the final month was 9.76% and the 10% target is now well within the control limits. Additionally, there were 5 months post-intervention where mortality was at or below 10%.

## Discussion

### Appropriate patient selection

Appropriate patient selection reduces NIV mortality.^5^, ^11^ The NCEPOD report found that NIV was inappropriate in 18.8% and two-thirds of those patients died.^5^ Prior to project commencement we agreed, across all sites, what we deemed as appropriate use of NIV; this was comfirmed as treatment initiation in patients with AHRF and a condition where there is an evidence base for NIV use, and where intubation or palliative care was not more appropriate. Clinicians retrospectively assessed if patients had been appropriately or inappropriately treated with NIV. The mortality rate improved when only including the appropriate group in analysis. Within the bundle and staff training, we therefore emphasised the importance of patient selection. Subsequently, staff confidence in identifying appropriate patients improved between surveys (Fig. 1).

### Early and effective treatment

Early NIV in AHRF reduces mortality.^5^, ^11^ We used ʻdecision to treatment within 60 min’ rather than ʻdoor to mask time’, as we included patients who were acidotic on admission and those with an initially normal pH developing AHRF later in their admission, with mortality for the latter group being higher.^12^ Staff training and the bundle emphasised early NIV initiation. Iterative feedback cycles revealed delays due to repeated trials of medical therapy and reluctance to commence NIV when acidosis was deemed ʻmild’. These issues were recognised and addressed with targeted staff education.

Insufficient pressure escalation causes prolonged acidosis and worsens outcomes. There is reluctance to escalate IPAP, especially among non-respiratory clinicians. In the NCEPOD report 45% of patients had an IPAP less than 20 cmH_2_O and 21% had no escalation of initial IPAP.^5^ We created the NIV5 for non-specialists, therefore a pragmatic minimum target IPAP of 20 cmH_2_O within 60 min was agreed with stakeholders to reduce the risk of insufficient ventilation and improve system reliability. We recognise that some patients, such as those with low BMI or neuromuscular disease, may achieve normalisation of pH at lower pressures. The need for individualisation of ventilation was therefore explored in staff education. However, our experience is that insufficient IPAP escalation is a more prevalent risk and was not reliably achieved in our project, therefore remained a key message in staff training.

### Standardisation of care

Commencing NIV can be intimidating, particularly for non-respiratory clinicians. We know from the NCEPOD report that most clinicians initiating NIV work in acute and emergency departments.^5^ The NIV5 bundle has established a standardised set of processes to reduce variability in practice and guide clinicians in initiating acute NIV. The bundle has been introduced across all hospitals in the region, helping provide institutional memory and familiarity for rotational staff.

### Staff confidence and competence

Staff education was delivered through multiple formats and the bundle was used as a framework for training. We believe the improved mortality during the project reflects both increased staff confidence and competence. We note there was a reduction in the number of staff completing the post-intervention questionnaire and suspect this was due to questionnaire fatigue.

### Challenges and limitations

Care bundles can be criticised as increasing tick-box, paperwork burden and oversimplification of complex processes. Relevant stakeholder engagement with ED and acute medicine was therefore critical. There was initially poor uptake of the bundle; however, compliance improved over time. Additionally, staff feedback indicated that the bundle acted as a cognitive aid increasing confidence in initiating NIV. Although no patient-identifiable data were shared, establishing data-sharing agreements between trusts was challenging. This approach proved beneficial, as feedback on combined mortality rates served to engage clinicians and focus training.

### The future

Our data reflect broader findings of the NCEPOD and BTS audits, that patients with severe acidosis are being managed in ward-based settings, contrary to guidance.^5^, ^8^ The recent BTS audit demonstrated that respiratory support units (RSUs) can reduce mortality.^8^ While ideally every hospital would be equipped with an RSU, such a significant change in infrastructure takes time. We demonstrated reduced mortality with a standardised care bundle and staff education, offering another way to improve mortality. As our bundle improves care in the first hours of NIV, it can be used before transfer to an RSU. Combining the two has the potential to reduce mortality further. Other future improvements could include earlier involvement of respiratory consultants and designated NIV teams or practitioners.^8^

To sustain improvements, we continue to measure the primary outcome and hold regular collaborative meetings, responding to change in addition to ongoing staff training.

## Conclusion

This project demonstrates that early NIV treatment delivered by confident, well-trained staff can improve outcomes. This was achieved by simplification and standardisation of the way that NIV is initiated in the first few hours.

## Ethics approval and consent to participate

No ethical approval was required for this project.

## Funding

The Health Innovation West of England supported this QI project extensively with project support/statisticians/data support and a financial contribution to an NIV patient video. Great Western Hospital, Swindon, Charitable funds provided £500 toward the NIV patient video production translation and BSL signing. Royal United Hospital, Bath, Charitable funds provided £500 toward the NIV patient video production translation and BSL signing.

## CRediT authorship contribution statement

**Rebecca Helen Mason**: Writing – review & editing, Conceptualization. **Mark C. Juniper:** Writing – review & editing, Conceptualization. **Peter W. Creber**: Writing – review & editing, Conceptualization. **Susie Beresford**: Methodlogy, Software, Formal analysis, Data curation. **Kirsty R. Ward**: Writing – review & editing, Conceptualization. **Christopher J. Michie**: Writing – review & editing, Conceptualization, Writing – original draft.

## Declaration of Competing Interest

The authors declare that they have no known competing financial interests or personal relationships that could have appeared to influence the work reported in this paper.
