# Supplementary figures and images for: A multi-centre quality improvement project to assess the impact of a standardised NIV care bundle on mortality outcomes in patients with acute type 2 respiratory failure
Source: Clin Med (Lond). 2026 May 6;26(3):100574. doi: 10.1016/j.clinme.2026.100574 (PMC13156728; doi:10.1016/j.clinme.2026.100574)

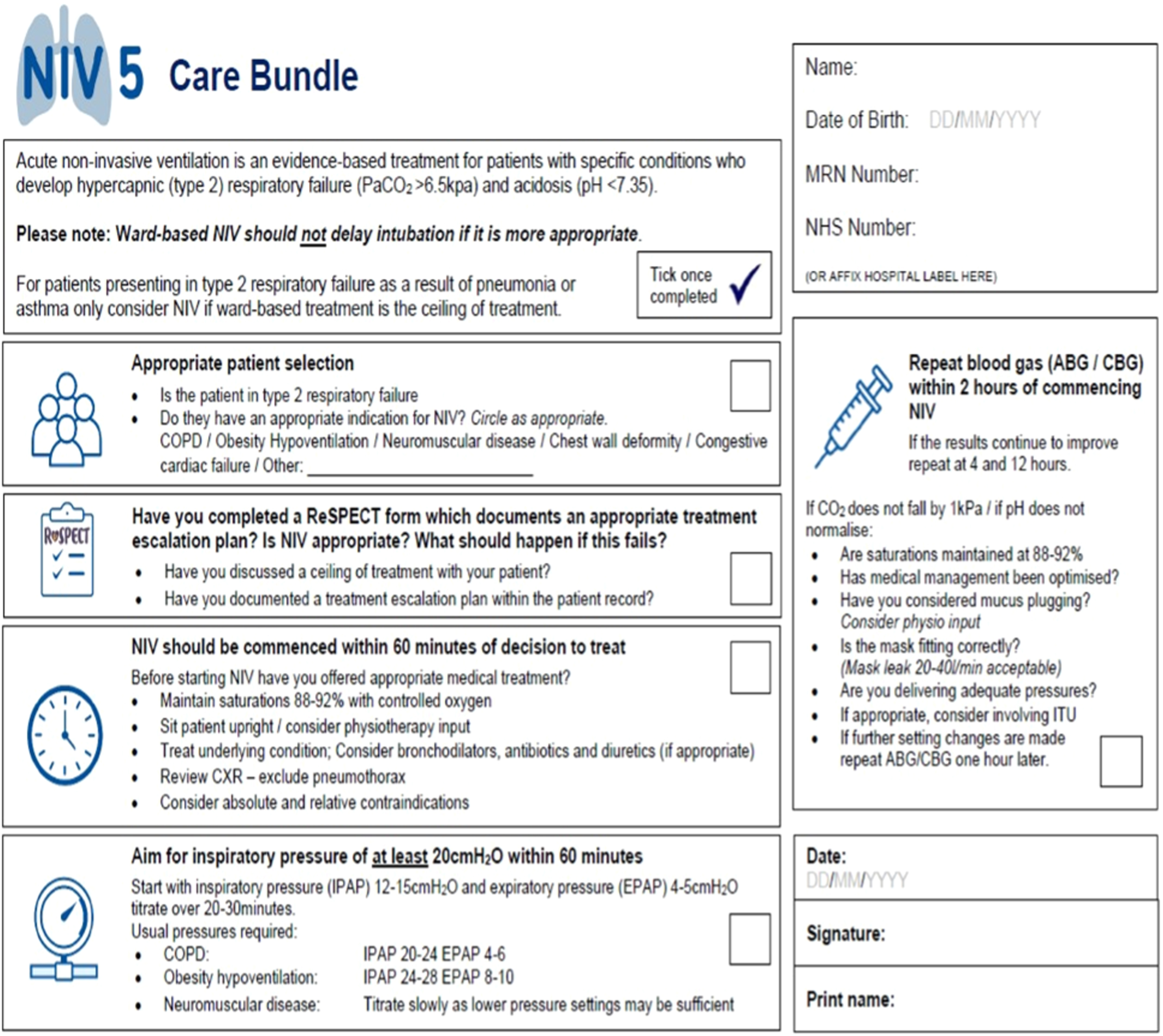

Supplement: Supplementary file 1 — Supplementary material [file mmc1.jpg]
